# ULTRASONOGRAPHY, SHEAR WAVE ELASTOGRAPHY AND LIVER HISTOLOGY FOR THE DIFFERENTIAL DIAGNOSIS BETWEEN BILIARY ATRESIA AND OTHER CAUSES OF CHOLESTASIS

**DOI:** 10.1590/S0004-2803.24612025-048

**Published:** 2026-03-02

**Authors:** Thais Costa Nascentes QUEIROZ, Rogério Auguto PINTO-SILVA, Eleonora Druve Tavares FAGUNDES, Guilherme Domingues FERREIRA, Ana Carolina Domingues FERREIRA, Alexandre Rodrigues FERREIRA

**Affiliations:** 1 Universidade Federal de Minas Gerais, Hospital de Clínicas, Belo Horizonte, MG, Brasil.; 2 Universidade Federal de Minas Gerais, Faculdade de Medicina, Belo Horizonte, MG, Brasil.; 3 Faculdade de Ciências Médicas de Minas Gerais, Belo Horizonte, MG, Brasil.

**Keywords:** Biliary atresia, elastography, histology, liver, Atresia Biliar, elastografia, histologia, fígado

## Abstract

**Background:**

Biliary atresia (BA) is the leading cause of cholestatic jaundice in the first months of life. Liver stiffness measurement by shear wave elastography (2D-SWE) could help discriminate BA from other causes of cholestasis.

**Objectives:**

To assess the use of abdominal ultrasound with bidimensional shear wave elastography and liver histology to diagnose Biliary Atresia in cholestatic infants. To compare the use of elastography to estimate the stage of liver fibrosis with the histologic classification.

**Methods:**

Cholestatic infants younger than three months were divided into BA and non-BA groups (other than neonatal cholestasis). 2D-SWE measured liver stiffness, and fibrosis was measured by Metavir score. Receiver operator characteristic (ROC) curves were developed to assess whether the variables of liver stiffness could be used to identify patients with BA and the best cutoff values.

**Results:**

21 infants with BA and 26 non-BA were included, of which 53,2% were males. The triangular cord was seen in 15/21 (71.4%) of BA and 2/26 (7.7%) non-BA, *P*<0.0001. The median value of liver stiffness in the first group was 2.7 m/s (IQ 2.1/3.6) and 1.6m/s (IQ 1.2/2) in the second group, *P*<0.0001. The area under the ROC curve to predict BA was 0.85 (95%CI, 0.74-0.96; *P*<0.0001). The best cutoff value was 1.99 m/s with sensitivity 81% and specificity 73.1%. Patients with BA classified as F0-2 had mean liver stiffness values by the 2D-SWE of 1.8±0.2m/s, and F3-4, mean values of 3±0.8m/s, *P*=0.008.

**Conclusion:**

Ultrasound and histology contribute to distinguishing BA from other diagnoses. Liver elastography is a promising tool in the differential diagnosis between BA and other causes of cholestasis, allowing the degree of fibrosis to be estimated at diagnosis.

## HIGHLIGHTS

• The diagnostic performance of liver stiffness measurement by SWE changed with age. SWE in <45 days did not show significance in predicting BA. However, in <60 days it did show.

• SWE is a promising tool that allows estimating the degree of fibrosis in the diagnosis of biliary atresia.

## RESUMO

**Contexto:**

Atresia biliar (AB) é a principal causa de icterícia colestática nos primeiros meses de vida. A rigidez hepática avaliada pelo Shear wave elastography (2D-SWE) pode ajudar a discriminar a AB de outras causas de colestase.

**Objetivo:**

Avaliar a ultrassonografia abdominal com bidimensional shear wave elastography e histologia hepática em lactentes colestáticos para diagnóstico diferencial entre atresia biliar e outras causas de colestase em lactentes. Avaliar a associação da rigidez hepática por 2D-SWE em comparação à classificação histológica da fibrose.

**Métodos:**

Foram incluídos lactentes de até 3 meses de idade com colestase, divididos em 2 grupos: AB e não-AB (colestase de outras etiologias). Avaliada rigidez hepática por 2D-SWE e fibrose segundo classificação de Metavir. Foram construídas curvas receiver operator characteristic (ROC) para analisar se as variáveis de rigidez hepática poderiam ser utilizadas para identificação de pacientes com AB e os melhores pontos de corte.

**Resultados:**

Foram incluídos 21 lactentes com AB e 26 não-AB, sendo 53,2% do sexo masculino. Cordão triangular foi visto em 15/21 (71,4%) na AB e 2/26 (7,7%) não-AB, *P*<0,0001. A mediana dos valores de rigidez hepática no primeiro grupo foi de 2,7m/s (IQ 2,1/3,6) e no segundo de 1,6m/s (IQ 1,2/2), *P*<0,0001. Área sob a curva ROC para a rigidez hepática para predizer AB foi de 0,85 IC95% 0,74-0,96; *P*<0,0001). O melhor valor de ponto de corte foi de1,99 m/s, com sensibilidade de 81% e especificidade de 73.1%. Pacientes com AB que tiveram classificação F0-2, tiveram média de valores de rigidez hepática por 2D-SWE de 1,8±0,2m/s e os F3-4, média de 3±0,8m/s, *P*=0,008.

**Conclusão:**

Achados ultrassonográficos e histológicos auxiliam na diferenciação de AB e outros diagnósticos**.** Elastografia hepática é promissora no diagnóstico diferencial entre AB e outras causas de colestase, podendo dimensionar o grau de fibrose ao diagnóstico.

## INTRODUCTION

Biliary Atresia (BA) results from an inflammatory process that affects the intrahepatic and extrahepatic bile ducts, causing fibrosis and obliteration of the biliary tract and subsequent cirrhosis^1^. It is the main cause of cholestatic jaundice in the first months of age (25-40%)^2^ and the leading indication for liver transplantation in children^1^ ^,^ ^3^.

The diagnosis of BA should always be considered when cholestatic jaundice extends beyond the 14th day of life^4^. Its diagnosis is still a challenge, primarily based on the combination of clinical, laboratory, ultrasonography, and histologic evaluation.

Ultrasound diagnostic criteria have been described and enhanced, and those considered most suggestive are the presence of the triangular cord sign at the portal bifurcation and its thickness, the diameter of the hepatic artery and the portal vein, the absence of a gallbladder or its morphology when present and the absence of intrahepatic biliary tract dilation^5^ ^-^ ^7^.

Liver biopsy is still an important exam; however, it is invasive and carries risks of complications^8^. Less invasive and cheaper exams hold potential for early diagnosis and good correlation with the extent of fibrosis and allow an easy longitudinal evaluation^9^. Bidimensional Shear Wave Elastography (2D-SWE) is a relatively recent exam that aims to quantify the stage of liver fibrosis by measuring hepatic stiffness. It has presented good diagnostic effectiveness for BA during abdominal ultrasound^9^ ^,^ ^10^.

The present study aims to evaluate hepatic stiffness by 2D-SWE, ultrasonographic findings suggestive of BA, such as the triangular cord, and liver histology for the differential diagnosis between BA and other causes of cholestasis. It also aims to evaluate the association between the stage of fibrosis detected by elastography and liver biopsy.

## METHODS

### Patients and study design

This observational study enrolled infants aged less than three months with cholestasis evaluated at Hospital das Clínicas da UFMG (HC-UFMG) from 2017 to 2020. A non-probability sampling was conducted on patients referred to the HC-UFMG’s Paediatric Hepatology service to investigate the cause of cholestasis. Patients were divided into two groups: BA and non-BA (cholestasis of other etiologies). All patients from the BA group had their diagnosis confirmed based on the combination of findings from ultrasonography, histology and preoperative cholangiography.

### Variables and definitions

Clinical variables were evaluated: sex, age, weight at admission, and age at sonography examination; and laboratory variables: albumin, liver (AST - aspartate aminotransferase and ALT - alanine aminotransferase) and canalicular (GGT - gamma-glutamyl transferase) enzymes, bilirubin dosing (TB - total bilirubin and DB - direct bilirubin), platelets, and AST to Platelet Ratio Index (APRI score).

Sonographic variables were the presence of the triangular cord sign, (echogenic thickening of the anterior wall of portal vein bifurcation), atrophic or rudimentary gallbladder, and hepatic stiffness measured by 2D-SWE. The sonographic analysis was conducted by a single sonographer (RAPS) with extensive experience in the sonographic investigation of cholestasis. Complete ultrasound evaluation of the liver, biliary tract, and spleen was conducted before elastography measurements. Before the exam, all infants were required to fast for 3 to 4 hours. Sometimes, feeding was allowed during the exam to calm the infant. Elastography measurements of the first patients were performed using a Supersonic Aixplorer device v.8.5.02 and a linear 4-15 probe (Supersonic, Aix-en-Provence, France). Since January 2019, the exams were done in Aplio i600 using a linear probe i8C1 (Canon Medical Systems, Otawara, Tochigi, Japan). For the hepatic stiffness measurements, the probe was positioned intercostally in the right middle or anterior axillary line, with caution not to perform compression. Ten measurements were carried out within a 10 mm diameter box. The measurements were taken in kPa and m/s, discerning mean, standard deviation, median, and interquartile range (IQR). Afterward, the IQR was divided by the median. Reliable results can be considered when the IQR/median is less than 0.30 in kPa or less than 0.15 in m/s.

Histologic variables were stage of fibrosis determined and categorized by Metavir score^11^ (F0-2 absent, mild or moderate fibrosis and F3-4 moderate to marked fibrosis); presence or absence of ductular proliferation, portal fibrosis, and degenerative events; degree of gigantocellular proliferation (categorized in light, moderate or marked). The fragments of liver were obtained through percutaneous puncture using a 16 or 18-gauge biopsy needle, only samples with >10 portal tracts were considered for analysis. Each fragment was fixated in 10% saline formol and processed according to histology routine until incursion in paraffin; the paraffin blocks are submitted to microtome and, from each block, five levels containing escalated histologic cuts are obtained with 5.0 to 7.0 mm of thickness, which are stained in hematoxylin and eosin (HE), Gomori or Masson trichrome and PAS with and without diastase. The maximum time between elastography and biopsy was 7 days.

### Statistical analysis

Statistical analysis was performed using Statistical Package for Social Sciences (SPSS®) 20 (IBM, New York, USA) and MedCalc 16.8.4. The Shapiro-Wilk test was used for descriptive analysis; variables with normal distribution were described using average and standard deviation, and variables without normal distribution were described using median (25th-75th interquartile range). Categorical variables were described using absolute count and percentage. Pearson’s asymptotic chi-square test (up to 20% of the expected values less than 5 and 80% of the expected value greater than 5) and Pearson’s exact chi-squared test (more than 20% of the expected value less than 5) were used to compare categorical variables. Student’s t-test was used to compare quantitative variables with normal distribution and equal variance, and variance homogeneity was verified by the Levene test. Mann-Whitney test was used for variables without normal distribution.

Receiver operator characteristic (ROC) curve analyses were carried out to determine if hepatic stiffness variables (m/s) and (kPa) could be used to identify BA and its best cutoff values. The area under the curve (AUC) and CI95%, as well as sensitivity, specificity, and accuracy measurements were considered. A boxplot was elaborated to correlate hepatic stiffness measurements with the Metavir score for histology. The significance values were set to 5%.

### Ethical aspects

The UFMG Ethical Committee approved the present research (resolution n^o^ 77.0.203000-09).

## RESULTS

### Data analysis

During this study, 47 patients aged less than three months were seen at HC-UFMG due to cholestasis. 21 (44.7%) were diagnosed with BA and 26 (55.3%) with other causes of cholestasis (non-BA group). In the non-BA group, the primary diagnoses were multi-factorial cholestasis 10/26 (38.5%), idiopathic neonatal hepatitis (23.1%), Alagille Syndrome (7.7%), Alfa-1 antitrypsin deficiency (7.7%), cryptogenic cirrhosis (7.7%) and other diagnosis (15.4%). After comparing both groups, it was noted that BA patients were admitted with a higher medium weight than the non-BA group, *P*=0.02.

All patients were subjected to abdominal ultrasound, and the triangular cord sign was present in 15/21 (71.4%) of the BA group and 2/26 (7.7%) in non-BA, *P*<0.0001. An atrophic or rudimentary gallbladder was more frequent amongst the BA group, *P*<0.0001. The mean hepatic stiffness measured by dynamic elastography was statistically higher in the BA group, 19.7 kPa (2.7 m/s) x 8.5 kPa (1.6 m/s) in the non-BA group (*P*<0.0001).

Amongst laboratory findings, only GGT showed significance when compared between both groups, *P*=0.007 (Table 1).

**TABLE 1:** Descriptive analysis of clinical, laboratory and ultrasound findings in BA and non-BA patients.

Variables	BA	Non BA	*P*
	(n=21)	(n-26)	
Sex n (%)			
female	10(47.6)	12(46.2)	0.920^1^
male	11(52.4)	14(53.8)	
**Weight at admission (grams)**			
Média±SD*	**4559.7±1135.5**	**3646±12720.8**	**0.020** ^2^
Age at US n (%)			
<60 days	8(38.1)	13(50)	0.414^1^
≥60 days	13(61.9)	13(50)	
Age at US n (%)			
<45 days	4(19)	8(30.8)	0.360^1^
≥45 days	17(81)	18(69.2)	
**Triangular cord n (%)**			
**not visualized**	6(28.6)	24(92.3)	<0.0001^1^
**visualized**	15(71.4)	2(7.7)	
Gallbladder n (%)			
atrophic or rudimental	10(47.6)	0(0)	<0.0001^3^
normal	11(52.4)	26(100)	
Hepatic stiffness in SWE (kPa)			
**Median (Q1; Q3)**	**19.7(13.1; 40.2)**	**8.5(7.2; 12.4)**	**<0.0001** ^4^
**Hepatic stiffness in SWE (m/s)**			
**Median (Q1; Q3)**	**2.7(2.1; 3.6)**	**1.6(1.2; 2)**	**<0.0001** ^4^
TB (mg/dL)			
Median(Q1; Q3)	10.2(7.3; 13.4)	8.4(7.1; 10.8)	0.171^4^
DB (mg/dL)			
Median (Q1; Q3)	7.9(5.5; 10.8)	5.6(4.3; 8)	0.105^4^
AST (U/L)			
Median (Q1; Q3)	230(179; 415.5)	180(68.3; 352.8)	0.097^4^
ALT (U/L)			
Median (Q1; Q3)	149(105.5; 289.5)	121(75.5; 226)	0.247^4^
**GGT (U/L)**			
**Median (Q1; Q3)**	**544(213.5; 778)**	**200.5(98.5; 366.8)**	**0.007** ^4^
Albumin (g/dL) Mean±SD	3.4±0.6	3.7±0.8	0.163^2^
APRI SCORE			
Median (Q1; Q3)	1.7(0.9; 2.9)	1.7 (0.4; 3.2)	0.748^4^
Platelets (/mm^3^)			
Mean±SD	407.095±144.788	373.192±170.466	0.473^2^

^1^Asymptotic Pearson Chi-square test. ^2^T test for equal variances. ^3^Exact Pearson Chi-square test. ^4^Mann Whitney test. ALT: alanine aminotransferase. APRI score: platelet ratio index. AST: aspartate aminotransferase. BA: biliary atresia. DB: direct bilirubin. GGT: gamma-glutamyl transferase. Q1: 1º quartile. Q3: 3º quartile. TB: total bilirubin. SD: standard deviation. SWE: shear wave elastography. US: ultrasound.

**FIGURE 1:**
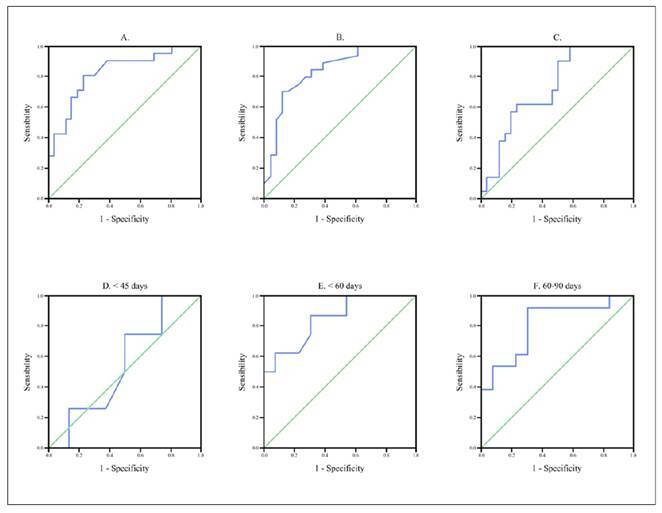
ROC to predict BA: A-SWE (kPa). B-SWE (m/s). C-GGT. D-SWE (kPa <45 days). E - (kPa <60 days). F - (kPa 60-90 days).

The best cutoff values for hepatic stiffness measurements to predict BA was 7.1 kPa with AUC 0.82 (95%CI, 0.71-0.95; *P*<0.0001), sensitivity 90.5% and specificity 61.5%, and accuracy 73.3%. When measured in m/s, the best cutoff value was >1.99m/s to predict BA, with an AUC 0.85 (95%CI, 0.74-0.96; *P*<0.0001), sensitivity 81% and specificity 73.1% and accuracy 76.6%. (Figure 1A and B ).

When analyzing age subgroups at the time of US, the hepatic stiffness shows no discrimination in predicting BA among infants up to <45 days. However, among children with <60 days, the hepatic stiffness showed good accuracy for predict BA; the best cutoff value was 8.95 kPa, AUC 0.85 (95%CI, 0.69-0.99; *P*=0.008), sensitivity 87.5% and specificity 69.2%. For 60-90 days of age, the best cutoff value was 12.05 kPa, with AUC 0.81 (95%CI, 0.69-0.98; *P*=0.007), sensitivity 92.3% and specificity 69.2% (Figure 1D, E and F ).

GGT analysis showed worse performance than hepatic stiffness; the best cutoff values was 167U/L (AUC 0.73; 95%CI, 0.59-0.87; *P*=0.007), sensitivity 90.5%, specificity 50%, and accuracy 70.2% (Figure 1C ).

Histologic analysis was performed in 24/47 (51.1%) of patients, of which 17/21 (80.9%) were in the BA group and 7/26 in the non-BA group. In 4 patients diagnosed with BA, a wedge instead of needle biopsy was performed during portoenterostomy and, therefore, were not considered for analysis of fibrosis. When comparing the histological findings, portal fibrosis and ductal proliferation were more common in BA patients (*P*=0.045 for both variables). Other variables, including degenerative events, gigantocellular transformation and stratified Metavir score (F0-2 and F3-4), were not different between the two groups.

**FIGURE 2:**
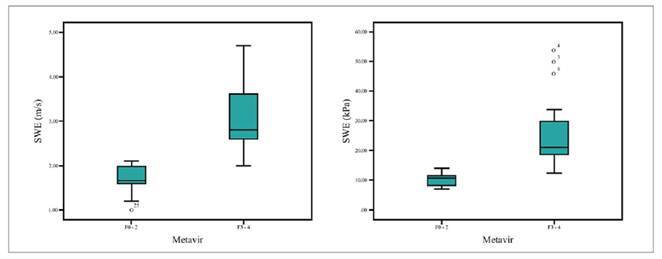
Boxplots for SWE (m/s) e SWE (kPa) variables according to metavir.

Liver fibrosis on biopsy was compared to clinical variables and hepatic stiffness measurements. APRI Score did not correlate to the extent of fibrosis in biopsy (Table 2). Hepatic stiffness in BA patients was statistically higher in the F3-F4 group in comparison to the F0-F2 group (23.8 kPa x 10.3 kPa, *P=*0.005) (Table 2). The box plots for the hepatic stiffness (m/s) and (kPa) variables confirm the higher values for patients with marked fibrosis or cirrhosis (Figure 2)

**TABLE 2:** Comparison of extent of fibrosis and clinical, laboratorial and elastography variables.

Metavir	F0-2	F3-4	*P* **value**
	(n=9)	(n=15)	
BA	4 (44.4)	13 (86.7)	0.061^1^
Non BA	5 (55.6)	2 (13.3)	
sex			
female	6 (66.7)	7 (46.7)	0.423^1^
male	3 (33.3)	8 (53.3)	
GGT (U/L)			
Median (Q1; Q3)	277 (134.5; 652.5)	690 (264; 993)	0.089^2^
APRI SCORE			
Median (Q1; Q3)	1.3 (0.8; 2.2)	1.7 (1; 2.6)	0.174^2^
Platelets (/mm^3^)			
Mean±SD	388.000±108.166	437.200±132.581	0.358^3^
**Hepatic stiffness measured by SWE (kPa) - BA**	**n=4**	**n=13**	
**Median (Q1; Q3)**	**10.3 (8.2; 13.1)**	**23.8 (17.1; 40.2)**	**0.005** ^2^
**Hepatic stiffness measured by SWE (m/s) - BA**	**n=4**	**n=13**	
**Mean±SD**	**1.8±0.2**	**3±0.8**	**0.008** ^3^

^1^Pearson’s exact chi-square test. ^2^Mann whitney test. ^3^T-test. APRI score: platelet ratio index. BA: biliary atresia. GGT: gamma-glutamyl transferase. Q1: 1º quartile. Q3: 3º quartile. TB: total bilirubin. SD: Standard deviation. SWE: shear wave elastography.

## DISCUSSION

Prompt differentiation between BA and other causes of cholestasis remains urgent, as early intervention improves outcomes promoting survival with native liver^2^. In the present study, we employed laboratory, histology, and ultrasound analysis, emphasizing elastography, to differentiate BA from other causes of cholestasis. The isolated analysis of these variables does not define BA diagnosis, but combining them may contribute to the medical team’s clinical judgment in the differential diagnosis of cholestasis.

Serum biomarker GGT showed a statistically significant difference, presenting higher values in BA patients. The best cutoff value to differentiate the diagnosis was 167 U/L (AUC=0.73), similar to the one found in Liu et al.^12^ study that analyzed 156 patients, categorized as BA (n=83) and non-BA (n=73) and found GGT ≥195.4 U/L (AUC de 0.82; 95%CI, 0.76-0.89; *P*<0.0001). GGT role in diagnosing BA varies widely, with accuracy ranging from 39.7% to 88%^12^ ^-^ ^16^. The present study found an accuracy of 70.2%, although values overlap between the groups, and a low GGT does not exclude BA.

Abdominal ultrasound has been used to evaluate newborns’ liver and biliary tract for many years. The presence of typical findings such as periportal echogenic thickening, morphological changes in the gallbladder or absent gallbladder, and findings suggestive of lateral defect or polysplenia syndrome, help differentiate BA from non-surgical causes of neonatal cholestasis^10^ ^,^ ^17^. However, these findings require great expertise from a sonographer and largely depend on the examiner’s experience. In the present study, we found a statistically significant difference associated with the presence of triangular cord and rudimental/atrophic gallbladder to differentiate BA from other causes of cholestasis.

Liver elastography is a well-established method for evaluating and following up on chronic liver disease in adults. It is gradually proving to be a reliable method for conducting pediatric cases, with studies^17^ ^-^ ^21^ demonstrating good accuracy in predicting the extent of liver fibrosis, even in early stages, which confirms its utility as a non-invasive method for detection and follow-up.

In a prospective study published in 2016, Wang et al.^22^ analyzed elastography data of 38 children diagnosed with BA submitted to Kasai surgery who had liver biopsy performed the day before the surgery, and compared to 17 children with neonatal hepatitis and 31 healthy controls. The hepatic stiffness value in the BA group was higher than in the other groups, and no statistical difference was found between the control group and the neonatal hepatitis group. There was excellent diagnostic precision (AUC=0.997), sensitivity 97.5%, and specificity 100%, with positive predictive value 100% and negative predictive value 96.9% to identify atresia. In the present study, we found AUC 0.82 and high sensitivity 90.5% but a low specificity 62.5%, which could be explained by the absence of a healthy control group and the presence of established cirrhosis amongst some patients in the non-BA group.

In a retrospective study published in 2015, Leschied et al.^23^ concluded that hepatic stiffness values are higher among infants with BA than among children with other causes of cholestasis, even when evaluated at the same age. This suggests that elastography could be specific indirect evidence of BA, just as the present study suggests.

Elastography performance increases with age. Liu et al.^12^ found AUC 0.74 for infants 15-30 days and 0.91 for those 30-45 days; amongst <15 days old patients, SWE was unable to differentiate BA from other etiologies. Shen et al.^13^ reported a comparable model after evaluating 282 patients with jaundice divided into three age groups: 0-60 days, 60-90 days, and 91-120 days. Hepatic stiffness cutoff value and area under the ROC curve both increased with age. When we analyzed hepatic stiffness in subgroups (<45 days, <60 days, and 60-90 days) in our study, we did not find significance among patients younger than 45 days, but we observed differences in the other subgroups. We did not categorize <30 days due to the smaller number of patients in this age group since cholestatic patients still arrive late in our service.

The main histologic findings that define BA are ductular proliferation leading to portal space expansion, biliary plugs in the biliary ductules associated with fibrosis, portal-portal bridge arrangements, hepatocyte ballooning degeneration, and gigantocellular transformation^3^. According to Russo et al.^24^, ductal proliferation and the absence of sinusoidal fibrosis are the histology findings that best predict BA. In the present study, ductal proliferation and portal fibrosis had statistically significant differences amongst BA x non-BA groups, which emphasizes the relevance of these findings to BA diagnosis.

The only previous method for staging liver fibrosis was histology. The advancement of elastography methods allows non-invasive evaluation with good diagnostic precision of liver fibrosis and its complications.

A meta-analysis published in 2022 demonstrated that elastography can differentiate mild (F0-2) from marked (F3-4) fibrosis, with 85% sensitivity and 81% specificity amongst children with BA^25^. Yoon et al.^26^ found a cutoff value of 17.3 kPa to predict advanced fibrosis F3-4 in 33 patients in preoperative Kasai surgery. Chen et al.^27^ suggest the cutoff of 13.0 kPa for F3 and 15.7 kPa for F4. In this study, hepatic stiffness by 2D-SWE showed better diagnostic performance in predicting the fibrosis stage than APRI score and other serum markers of fibrosis. Ahmad et al.^28^ assessed 21 infants with BA using 2D-SWE and liver biopsy before Kasai portoenterostomy and could verify that amongst patients presenting F2 or F3 Metavir fibrosis at biopsy, hepatic stiffness was well correlated to the stage of fibrosis, being 10.28 kPa in the F2 group and 14.49 kPa in the F3 group. None of the patients had mild fibrosis or cirrhosis. Therefore, it was impossible to determine cutoff values for these fibrosis stages. Our study used 2D-SWE to analyze hepatic stiffness values in BA patients categorized as F3-4 by liver biopsy. We found a median of 23.80 kPa that presented a statistical difference to F0-2 BA patients (10.3 kPa). Therefore, their values are different to our findings, perhaps because they don’t have cirrhotic patients.

Previous studies^27^ ^,^ ^29^ attempted to assess the APRI score as a tool to predict fibrosis in BA, but they did not find a good correlation, as our study did. An Indian research assessed 48 infants with non-BA cholestasis, with a mean age of 3.5 months, and divided them according to the Metavir score: 32 F0, 10 F1-2, and 6 F4. The APRI score was performed at the same time as the biopsy, and it was not effective in measuring fibrosis or cirrhosis^30^.

This study presents several limitations, such as not performing histological analysis in all participants. Also, among patients with BA, wedge biopsies were excluded from the analysis because the fragment is subcapsular and may overestimate liver fibrosis. In addition, several patients with other causes of cholestasis were not submitted for biopsy because they already had an established diagnosis by other methods, and the histological analysis would not change the conduction of their cases.

However, besides being conducted in a single center, our sample of 47 patients with cholestasis, 21 diagnosed with BA, all evaluated by bidimensional elastography, is relevant for comparison with other published cohorts. The results corroborate the available international published data.

## CONCLUSION

The present study demonstrates that ultrasound findings and hepatic stiffness measurements help differentiate BA from other causes of cholestasis.

Elastography methods are relatively recent and have been greatly optimized in the last two decades. The results are encouraging in pediatric age groups, so 2D-SWE should be included in the investigation and follow-up of pediatric chronic liver disease.

## Data Availability

Data-available-upon-request
